# Weaker Inter-hemispheric and Local Functional Connectivity of the Somatomotor Cortex During a Motor Skill Acquisition Is Associated With Better Learning

**DOI:** 10.3389/fneur.2019.01242

**Published:** 2019-11-27

**Authors:** Ella Gabitov, Ovidiu Lungu, Geneviève Albouy, Julien Doyon

**Affiliations:** ^1^McConnell Brain Imaging Center, Montreal Neurological Institute, Montreal, QC, Canada; ^2^Functional Neuroimaging Unit, Centre de recherche de l'Institut universitaire de gériatrie de Montréal, Montreal, QC, Canada; ^3^Département de Psychiatrie et d'Addictologie, Université de Montréal, Montreal, QC, Canada; ^4^Movement Control and Neuroplasticity Research Group, Department of Movement Sciences, KU Leuven, Leuven, Belgium

**Keywords:** motor cortex, motor learning, motor sequence, memory representation, functional connectivity, fMRI—functional magnetic resonance imaging, resting state, task activation

## Abstract

Recently, an increasing interest in investigating interactions between brain regions using functional connectivity (FC) methods has shifted the initial focus of cognitive neuroimaging research from localizing functional circuits based on task activation to mapping brain networks based on intrinsic FC dynamics. Leveraging the advantages of the latter approach, it has been shown that despite primarily invariant intrinsic organization of the large-scale functional networks, interactions between and within these networks significantly differ between various behavioral and cognitive states. These differences presumably indicate transient reconfiguration of functional connections—an instantaneous process that flexibly mediates and calibrates human behavior according to momentary demands of the environment. Nevertheless, the specificity of these reconfigured FC patterns to the task at hand and their relevance to adaptive processes during learning remain elusive. To address this knowledge gap, we investigated (1) to what extent FC within the somatomotor network is reconfigured during motor skill practice, and (2) how these changes are related to learning. We applied a seed-driven FC approach to data collected during a continuous task-free condition, so-called resting state, and during a motor sequence learning task using functional magnetic resonance imaging. During the task, participants repeatedly performed a short five-element sequence with their non-dominant (left) hand. As predicted, such unimanual sequence production was associated with lateralized activation of the right somatomotor cortex (SMC). Using this “active” region as a seed, here we show that unimanual performance of the motor sequence relies on functional segregation between the two SMC and selective integration between the “active” SMC and supplementary motor area. Whereas, greater segregation between the two SMC was associated with gains in performance rate, greater segregation within the “active” SMC itself was associated with more consistent performance by the end of training. Nether the resting-state FC patterns within the somatomotor network nor their relative modulation by the task state predicted these behavioral benefits of learning. Our results suggest that task-induced FC changes reflect reconfiguration of the connectivity patterns within the somatomotor network rather than a simple amplification or silencing of its intrinsic dynamics. Such reconfiguration not only supports motor behavior but may also predict learning.

## Introduction

The neural basis of high dimensionality (e.g., a large repertoire of actions that can be performed in various ways) and adaptability of human behavior has been extensively studied with functional magnetic resonance imaging (fMRI) (1, 2). Using this technological approach, the brain-behavior relationships have been primarily investigated by localizing task-activated brain regions, i.e., areas that exhibit significant increases in mean blood-oxygenated-level-dependent (BOLD) fMRI signal during tasks compared to rest or control conditions [for reviews, please see (3, 4)]. Over the past decade, however, there has been an exponential increase in the number of studies investigating spontaneous hemodynamic activity measured at rest with fMRI; that is, while participants lie quietly in the scanner without any explicit task or stimulus. In fact, assessing correlations of BOLD signal between brain regions during this resting state—a method referred as functional connectivity (FC) (5)—has proven to be a valuable technique for mapping functional networks, including the somatomotor system (6–11). A highly synchronized neural activity between distributed brain regions forming functional networks has been repeatedly demonstrated not only at rest but also during various tasks indicating their resilience to the behavioral or cognitive context (12–15). Together with the observation that spontaneous fluctuations in neural activity account for variability in task-evoked activations and associated behaviors (16–18), such findings lend support to the notion that functional networks in the brain are primarily invariant across behavioral states, whereas momentary demands of the environment play only a modulatory role in their intrinsic functions (19). As such, this view suggests that the functional ability and processing capacity of the brain can be inferred based on FC dynamics during the resting state, meaning that these intrinsic dynamics not only reflect unceasing intrinsically synchronized activity patterns, which are constrained by neuro-anatomical connections (20), but they also determine task-evoked activation and behavior (15, 21–25).

Recently, however, it has become clear that, despite the state-invariant, intrinsic organization of the large-scale functional networks, interactions between and within these networks during the task state significantly differ from their interactions during the resting state (26, 27). Such dissociation between the two states is expressed by rather complex pattern of FC changes, even during simple activities such as passive movie watching (26, 28), with some connections being significantly weakened, whereas others strengthened or unchanged. Although these changes are relatively small, in terms of their magnitude, it has been argued that at least some of them reflect reconfiguration of the functional neural connections rather than a simple amplification or silencing of the intrinsic brain dynamics. This idea of rapid reconfiguration is supported by previous work showing that some of the task-induced changes in the individual whole-brain FC patterns are specific to the ongoing task, hence allowing to accurately decode the type of cognitive processing imposed by such task (29, 30). Moreover, some of those transient FC patterns are related to individual differences in performance levels, suggesting that they are relevant to behavior (27, 31–38). Thus, despite a mainly preserved intrinsic large-scale FC topography across behavioral states, some transient changes in FC on a smaller scale, as captured with the BOLD-fMRI signal, may be the ones that grant humans the ability to flexibly adapt their behavior according to the task at hand (13, 14).

Yet, the relevance of task-induced changes in FC to specific behavior and adaptive processes during learning remains elusive. For instance, it has been shown that the FC strength between and within functional networks increases with task complexity, greater attentional demands, and better performance levels (31, 38–40), but may decrease with learning, which would indicate diminished cognitive control and sensory input dependency to enable automaticity (36, 41). It is worth noting that changes in FC are not limited to task-activated regions and may be dictated by the region's functional connectivity profile (i.e., a relative number of within- and between-network connections) (13), and the level of information processing (e.g., primary vs. multimodal associative areas) (42). Specifically, primary sensory and motor circuits have been found to be particularly prone to change their FC patterns when the brain is engaged in the task, as compared to the resting state. However, our current understanding of these dynamics under specific conditions and their potential role in learning is rather limited.

In the current study we sought to assess the extent to which FC of the somatomotor network is reconfigured by the task state and how these changes support motor task execution and learning. The hypothesis that some aspects of learning are associated with the FC of the somatomotor network during the resting state was also tested. To this end, we applied FC analyses to data from the fMRI experiment conducted by Albouy et al. (43), who scanned participants during both the continuous resting state period and a motor sequence task. During the task, participants repeatedly performed a short five-element sequence using their non-dominant (left) hand. Prior to the task, they were asked to memorize the sequence, i.e., five digits in the predetermined order—this amount of information is within the normal working memory capacity (44) and is easily remembered. In that way, during the task, participants were able to reproduce the sequence continuously in a self-paced manner without relying on any external cue or input. Also, no feedback was provided at any time during the actual performance.

This approach suits well our goal to investigate FC dynamics within the somatomotor network during motor execution and learning for several reasons. First, this version of the motor sequence learning task has been widely used to probe motor executive function, and is thought to engage the somatomotor network in relative isolation from the rest of the brain (31, 38). The segregation of the somatomotor network from other functional networks not only underlies actual motor sequence production, but is also associated with higher performance levels and better learning (23, 31, 41, 45). This suggests that specialized regions within this low-level network may contain dedicated neural populations that encode and represent motor sequences (46–48). Second, the stimulus-free mode of performance and the continuous nature of the task minimize attentional and cognitive load, thereby allowing greater isolation of the endogenous processes within the somatomotor network. This design is also advantageous for separation between the task-evoked activation and task-based FC patterns—an issue that is inherently present during stimulus-driven and event-related paradigms (49). Finally, the unimanual motor sequence production allowed us to compare FC dynamics between the two somatomotor cortices (SMC) that have highly coherent intrinsic activity but are differentially recruited during the task and, therefore, may differentially contribute to learning (50, 51). Such focus on the FC dynamics within the somatomotor network will provide novel insights into the type and level of knowledge represented within this primary circuit—a topic that has been debated for several decades but still remains controversial (46, 48, 52, 53).

Operationally, we refer to increases in FC strength as evidence for greater functional integration and information sharing, whereas decreases most likely reflect more segregated processing. Both processes may act in parallel and operate on multiple spatial scales affecting FC strength between networks, between regions within the same network, or between neural populations within the same region. Some of these changes, however, may also reflect reduction of correlated noise. Animal studies suggest that stimulus-driven noise reduction is a general property of the brain (54). It contributes to overall stabilization of functional circuits when the brain is engaged in information processing but lacks specificity and, by itself, does not improve fidelity of neural encoding (55, 56).

Using a seed-driven approach with the seed ROI within the SMC contralateral to the performing hand (i.e., the “active” SMC), our analyses were primarily focused on changes in FC within this task-activated region itself as well as between the two SMC. In addition, significant changes in FC with the supplementary motor area (SMA)—a region presumably involved in sequence representation across multiple domains (57)—are also reported. Similar to the SMC, the SMA contains somatotopic information (58, 59) and is part of the somatomotor network (11, 15, 60, 61). Specifically, we sought to distinguish between FC dynamics associated with (1) selective engagement of task-relevant neural representations during motor performance (62, 63) and (2) selective stabilization of these representations during practice (64)—two experience-driven processes proposed by animal studies. Clear behavioral consequences of the execution of the motor sequence task provide a reliable basis to assume that changes in FC between the resting and task states will capture reorganization within the somatomotor network relevant to motor performance. It is also well-established that repeated experience with the motor sequence results in faster and more stable performance (46, 47, 52, 65–68), thereby providing reliable and testable behavioral correlates of learning at the level of action execution and action selection (69).

## Materials and Methods

### Ethics Statement

All participants gave their written informed consent to take part in the study, which was approved by the Research ethics board of the RNQ (Regroupement Neuroimagerie Québec). All procedures were in accordance with the approved guidelines and regulations. Participants were compensated for their participation.

### Participants

The current report is based on the analyses of data collected during the initial resting state scan and the training session from a previous fMRI experiment published elsewhere (43). The sample included 55 healthy young right-handed (70) volunteers (mean age: 24.1 ± 3.5 years, 34 females) who were recruited by local advertisements to participate in the study. Participants were included in the study if they reported no history of medical, neurological or psychiatric disease. None of them were taking medications at the time of testing. All participants had a normal quality of sleep, as assessed by the Pittsburgh Sleep Quality Index questionnaire (71) and the St. Mary Hospital questionnaire (72). Also, none of the participants received formal training as a musician or as a typist.

In addition to the above-mentioned exclusion/inclusion criteria, we have also removed some participants' data from the analysis based on their performance. As such, one participant was excluded because his initial performance rate was slower than the group average by more than three standard deviations indicating a significantly lower general ability to use the keypad (participant's and group average time to complete the first three training blocks: 56.34 and 28.94 ± 8.01 s, respectively). Two additional participants showed very low accuracy levels by the end of training with only six correctly performed and completed sequences or less (out of the 12 repetitions of the sequence) during each of the last three blocks. Five others showed degraded performance levels by the end of training with slower tapping rate during the last than during the first three blocks. Poor accuracy and decreased performance rate below its initial levels by the end of training may indicate loss of interest or attentional biases that are out of the scope of the current study. Finally, one participant had excessive head movements and one participant did not have resting state data. Consequently, a total of 45 subjects, out of 55, were included in the analyses.

### Overall Experimental Design

All scanning runs were performed using functional magnetic resonance imaging (fMRI) while participants were lying supine in the scanner. First, participants underwent a resting state scan (6 min 40 sec) keeping their eyes open and looking at the fixation cross. They were asked to remain still and “not to think about anything in particular.” In that way, intrinsic activity during the resting state was not affected by the experience with the motor sequence task *per se* (73, 74). Next, while still in the scanner, participants received instructions about the motor sequence task (see below) and were scanned again while being trained on this procedural paradigm.

### Motor Sequence Task

**Figure 1:**
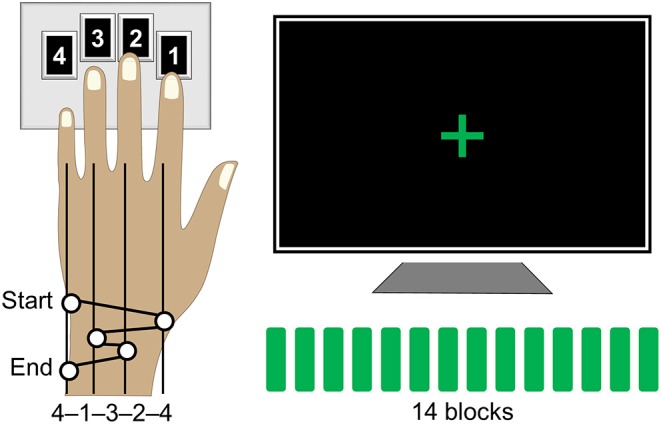
Motor sequence task. Participants were instructed to tap a five-element sequence (4-1-3-2-4) on a keypad using their left hand. The session consisted of 14 successive performance blocks with 60 keypresses each, equivalent to 12 repetitions of the sequence, separated by 15-s periods of rest. During performance blocks, participants were asked to look at the fixation cross and to tap the sequence repeatedly “as fast and with as few errors as possible.” A change in color of the fixation cross, from red to green and from green to red, indicated the beginning and the end of each performance block, respectively.

The motor sequence task was designed according to a paradigm that has been widely employed to study procedural memories in humans since its development (46) and was programmed in Matlab R2014a (The Mathworks, Inc., Natick, MA) using Cogent 2000 developed by the Cogent 2000 team at the FIL and the ICN and Cogent Graphics developed by John Romaya at the LON at the Wellcome Department of Imaging Neuroscience (http://www.vislab.ucl.ac.uk/cogent_2000.php). Training on this task required participants to tap a five-element sequence of finger movements on a keypad using their non-dominant (left) hand (Figure 1). The sequence (4-1-3-2-4) was introduced to participants using the numbers from 1 to 4 that corresponded to the four fingers of their left hand (excluding the thumb) from the index to the little finger, respectively. Participants received a full explicit introduction of the sequence and were asked to memorize it. The training session was initiated only after the sequence was reproduced three times in a row, without any error. During the actual training, participants were asked to look at the fixation cross and to tap the memorized sequence repeatedly “as fast and with as few errors as possible.” In case of occasional errors, they were instructed “to continue with the task from the beginning of the sequence.” No feedback was provided to the participants about their performance at any time of the experiment. The training session consisted of 14 successive blocks of practice with 60 keypresses within each block, i.e., equivalent to 12 repetitions of the sequence, and 15-s periods of rest between the blocks. Thus, the duration of the training blocks varied between participants as a function of their performance rate. Furthermore, participants developed faster performance rate spending less time to complete each block as training progressed. During the rest periods, participants were instructed to remain still and look at the fixation cross. A change in color of the fixation cross, from red to green and from green to red, indicated the beginning (“GO” cue) and the end (“STOP” cue) of each training block, respectively. Participants' performance was recorded by saving the code-number (i.e., 1, 2, 3, or 4) and time of each keypress.

### Behavioral Data Analyses

It has been consistently shown that experience with explicitly known motor sequences is associated with substantial changes in performance rate while the number of errors is extremely low (43, 52, 65, 66, 75). In line with these observations, the number of errors in the current sample of participants was indeed very low with 0.75 ± 0.08 errors per block (mean ± s.e.m.; an error corresponding to all, i.e., one or more, keys comprising one unsuccessful attempt/trial to perform the sequence—the initiation of each trial was determined by the first two elements within the sequence, i.e., 4-1…, and included these and following keypresses till the next trial; all incorrect keys following correctly performed and completed sequences till the next trial were also considered as an error). Therefore, performance levels were assessed using a measure reflecting performance rate, i.e., the time (duration in sec) per block spent executing the motor sequence task (43, 75).

**Figure 2:**
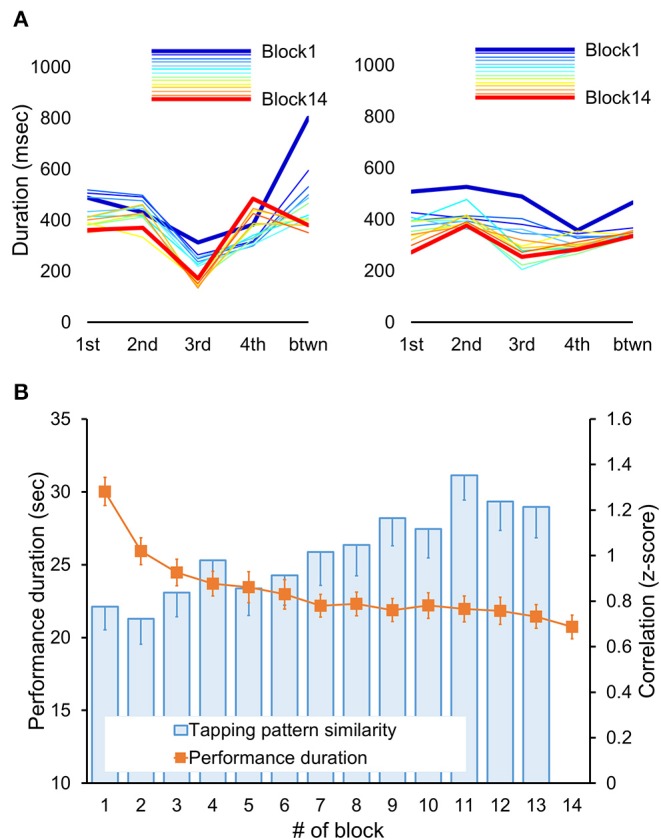
Behavioral results. **(A)** Tapping patterns, i.e., patterns of inter-keypress intervals, for each performance block (Block 1–14) are shown for two representative subjects. Each line connects data points representing mean duration (i.e., inter-keypress interval) for each of four possible transitions between successive elements within a sequence (from 1st to 4th) plus an additional transition between sequences (btwn) for each block. Thus, the shape of each line depicts an individual tapping pattern for a single block. Note, that the initial tapping pattern and its changes throughout the training differed between participants. With practice, the tapping pattern became progressively more similar to the one generated during the last training block (Block 14, red line). Yet, such increased similarity does not necessarily imply faster performance rate. **(B)** Performance duration and degree of tapping pattern similarity averaged across participants are shown for each performance block. The duration of each performance period was assessed in seconds starting from the first keypress following the “GO” cue. The degree of tapping pattern similarity to the tapping pattern formed by the end of training (i.e., during block 14) was assessed based on normalized Pearson's correlation coefficients using the Fisher's z-transformation. These coefficients were calculated for each block (blocks 1–13). Error bars represent standard error of the mean (s.e.m.). Fast improvement in performance speed, as indicated by significant decreases in performance duration across training blocks (orange markers), was also paralleled by significant changes in the tapping pattern so that its similarity to the tapping pattern formed by the end of training significantly increased (blue columns).

In addition to the development of faster performance, motor sequence learning also involves the formation of a novel tapping rhythm or pattern to generate the same sequence of movements (76). The tapping pattern, which is defined here as the relative temporal spacing between consecutive keypresses, may vary in the beginning but stabilizes by the end of the initial training (75). To assess experience-driven changes in that pattern, we used the same approach as the one published in our previous reports (75, 77). This approach estimates individual changes in the tapping pattern using correlation coefficients, thereby allowing to account for the inter-subject differences in the overall performance rate and the tapping pattern variability (68, 76, 78). To do so, we first extracted inter-keypress intervals, i.e., durations between successive keypresses, within and between all correctly performed and completed sequences separately for each performance block (Figure 2A). Next, these intervals were averaged according to their position within and between sequence repetitions in each block; values that were two standard deviations away from their corresponding mean were excluded. This procedure resulted in 14 five-element vectors (one for each block) representing individual tapping patterns of the sequence throughout the training. Finally, changes in these patterns were assessed using Fisher's *z*-transformed Pearson's correlation coefficients. These coefficients were calculated for blocks 1–13 using the tapping pattern generated during the last block as a reference. Thus, these correlation coefficients indicated the degree of similarity to the tapping pattern formed by the end of training. This measure is sensitive to the relative differences between successive keypresses so that higher values correspond to greater pattern similarity, i.e., greater consistency, and vice versa. However, it does not directly reflect changes in the overall performance rate. Furthermore, the correlation coefficients are sensitive to dynamic changes in the tapping pattern independently of its specific characteristics, such as shape and chunks, allowing valid comparisons at the group level without making any assumption in that regard.

Individual measures, reflecting performance rate and the degree of tapping pattern similarity, were analyzed using Statistical Package for the Social Sciences (SPSS Statistics for Windows, Version 24.0; IBM Corp., Armonk, NY). The analyses were run separately for each measure using repeated measures Analysis of Variance (ANOVA) with *block* as a within-subject factor. The results were corrected for non-sphericity violation using the Greenhouse-Geisser adjustment, when appropriate. We also calculated individual training-related gains in performance rate and the degree of tapping pattern similarity by averaging performance duration and correlation coefficients across the last six blocks. The former values were converted into percents relative to the mean performance duration during the first six blocks to account for inter-subject differences in the initial performance rate. These values were then used as covariates in analyses of functional connectivity patterns (see below).

### fMRI Data Acquisition

The fMRI time-series were acquired using a 3.0 T TIM TRIO scanner system (Siemens, Erlangen, Germany), equipped with a 32-channel head coil. T2^*^-weighted axial fMRI images sensitive to change in the BOLD signal were obtained with a gradient echo-planar sequence using interleaved acquisition mode in ascending direction (TR = 2.65 s, TE = 30 ms, FA = 90°, FoV = 220 × 220 mm^2^, matrix size = 64 × 64 × 43, voxel size = 3.4 × 3.4 × 3 mm^3^, 10% inter-slice gap). T1-weighted sagittal 3D MP-RAGE structural images were also obtained (TR = 2.30 s, TE = 2.98 ms, TI = 900 ms, FA = 9°, FoV = 256 × 256 mm^2^, matrix size = 256 × 256 × 176, voxel size = 1 × 1 × 1 mm^3^).

### fMRI Data Preprocessing

Both structural and functional images were converted to the Neuroimaging Informatics Technology Initiative (NIfTI) format using MRIcron (University of South Carolina). Preprocessing of the data was carried out with SPM12 (http://www.fil.ion.ucl.ac.uk/spm/software/spm12/; Wellcome Trust Center for Neuroimaging, London, UK) operating under Matlab R2014a (The Mathworks, Inc., Natick, MA). Functional volumes were realigned using a least squares approach and a six-parameter (rigid body) spatial transformation to correct for a movement-related variance. Following segmentation and skull-stripping of the structural data, functional images were coregistered to the individual skull-stripped 3-D anatomical image and normalized to the Montreal Neurological Institute (MNI) space using parameters obtained from the segmentation procedure. The normalized functional images were resampled to voxel dimensions of 3 mm^3^ and spatially smoothed with an isotropic Gaussian kernel with a full-width at half-maximum (FWHM) of 6 mm to improve the signal-to-noise ratio. Head motion artifact detection was also applied using the Artifact Detection Tools (79) (normalized *z*-threshold = 5, movement threshold = 0.9 mm).

### Task-Induced Changes in Activity

Task-induced changes in brain activity were assessed on the preprocessed task-related fMRI images using a general linear model (GLM) approach implemented in SPM12. This approach was applied on the preprocessed fMRI images acquired during scanning of the motor task. Statistical analyses of fMRI time-series consisted of a two-stage summary statistics model (80). In the first stage, BOLD signal changes were estimated for each subject using a fixed-effect GLM. A covariate of interest for performance periods was modeled as a boxcar function, time-locked to the onset and duration of each block, convolved with the canonical hemodynamic response function (HRF). Volumes with motion artifacts were ignored using nuisance regression. A high-pass filter of 128 s was used to remove low-frequency noise. Serial correlations in fMRI signal were estimated through a restricted maximum likelihood (ReML) algorithm using a first-order autoregressive plus white noise model. Following parameter estimation, a linear contrast was defined to test the mean effect of performance blocks relative to the rest period.

In the second stage, the resulting individual contrast images (*t*-maps) were carried forward to the random effects GLM analysis to assess the consistency of the effect between subjects. The statistical inferences were done at the group level using a one-sample *t*-test. The resulting group activation map was thresholded at *p* ≤ 0.05 (two-tailed) using peak-level family-wise error (FWE) correction over the entire brain and overlaid on the mean structural image of all participants using Functional Imaging Visualization Environment toolbox for SPM (FIVE, http://mrtools.mgh.harvard.edu).

### Regions of Interest

A main region of interest (ROI), which was also used as a seed for the FC analyses, was defined within the primary somatomotor cortex (SMC) significantly activated during the task as compared to rest. All participants used their left hand to perform the sequence and, therefore, activation within the SMC was strongly lateralized to the right hemisphere. The ROI within this “active” SMC was defined as a sphere (*r* = 6 mm) centered at the nearest local activation maximum to the knob of the precentral gyrus, that is, the motor hand area (81). Due to the close proximity between the motor and somatosensory cortices and their simultaneous activation during the motor sequence task we refer to this region as a somatomotor hand area throughout the manuscript. An ROI within the left (“passive”) SMC was also defined in a similar way, in terms of its size and proximity to the hand knob, but using the task-related functional connectivity map of the “active” SMC (i.e., the seed; see below).

### Functional Connectivity Analyses

Analyses of functional connectivity (FC) patterns were performed on the preprocessed functional images acquired during the resting and task state using the Functional Connectivity Toolbox (Conn) for SPM (82). FC patterns were assessed using a seed-driven approach.

Prior to the FC analysis, the data underwent additional temporal preprocessing. We applied a component-based noise correction method (CompCor) (83) implemented in Conn to extract five principal components derived from the white matter and cerebrospinal fluid. These components were entered as temporal confounding factors along with the detected volumes with motion artifacts. For the time-series acquired during the training on the motor sequence task, the main effect of performance blocks convolved with the canonical HRF and the corresponding first-derivative terms were included as additional confounds. All confounding factors were removed from the time-series using linear regression. Finally, the resulting residual BOLD time series were also high-pass filtered (0.008 Hz < f).

Individual maps of FC patterns were generated by analyzing the resting state time-series (6 min 40 s, 150 volumes) and the time-series acquired while participants were performing the motor sequence task, separately. The overall time spent on actual performance varied between participants as a function of their performance rate. On average, they spent 5 min and 25 s (123 volumes) practicing the sequence; the total performance time of the fastest and slowest participant being 3 min 42 s (84 volumes) and 10 min 15 s (232 volumes), respectively. The performance periods were separated from the interleaved periods of rest by including a regressor related to performance blocks. To take into account the hemodynamic delay, this regressor was convolved with a canonical HRF and rectified. Thus, the task-based FC analyses were performed on the data acquired during continuous periods of actual performance leaving out the rest. In that way, FC measures reflected interaction between brain regions during the task state that was separated from the task-evoked activation (i.e., global changes in signal from rest to task and vice-versa) or signal fluctuations during interleaved periods of rest.

FC analyses were performed using the ROI within the “active” SMC as a seed. Individual FC maps were generated by estimating Fisher's *z*-transformed Pearson's correlation coefficients between the BOLD signal averaged across voxels within the seed region and that at every voxel in the brain.

The individual maps were introduced into a second level GLM analyses to obtain group-level estimates. The statistical inferences of the resting state and task-based FC patterns were done at the group level using a one-sample *t*-test. Task-induced changes were assessed by contrasting statistical maps between the two states ([Task state]—[Resting state]). The resulting maps were thresholded at *p* ≤ 0.001 (two-tailed) and overlaid on the mean structural image of all participants using FIVE. For the 3D visualization, maps were projected on the inflated mean cortical surface of all participants using a surface display implemented in Conn.

### Regression Analyses

In addition to the second-level analyses to obtain the group-level estimates described above, regression analyses with individuals' behavioral measures as covariates of interest were also performed. This approach allowed us to test for regions where FC strength with the “active” SMC was associated with individual training-induced changes in performance. These analyses were run separately for each performance measure (i.e., gains in performance rate and the degree of tapping pattern similarity) using the task-based FC maps. Possible relationships between the behavioral measures and the FC during the resting state, as well as its relative changes induced by the task state were also tested. Statistical inferences were made at the peak-level using family-wise error correction (FWE) over a small volume of interest. The volumes of interest were defined as spheres (*r* = 10 mm) around a center of each ROI. Statistics for clusters that survived a cluster-level extent threshold of *p* < 0.05 following a peak-level threshold of *p* < 0.005 (two-tailed) is also reported. The specificity of associations between FC values and learning measures to the behavioral state was tested as *post-hoc* comparisons between correlations from dependent samples using an online calculator (https://www.psychometrica.de).

## Results

### Behavioral Results

The time to complete each training block (i.e., performance duration) and the degree of tapping pattern similarity to the one attained by the end of training are shown in Figure 2B. Training-related changes in performance were assessed using repeated measures ANOVA with *block* as a within-subject factor. As expected, training on the motor sequence task led to a faster performance as indicated by a significant effect of *block* [*F*_(6.70, 294.43)_ = 53.49, *p* < 0.001]. On average, performance duration decreased from 30.03 ± 0.97 to 20.73 ± 0.814 s (mean ± s.e.m. of the first and the last training block, respectively). These robust gains in performance rate were paralleled by significant changes in the subjects' tapping pattern as indicated by a significant effect of *block* [*F*_(5.92, 260.31)_ = 4.76, *p* < 0.001] on the correlation coefficients between the tapping patterns of the last training block and each of the other training blocks. The degree of similarity to the tapping pattern generated during the last training block increased from 0.78 ± 0.10 to 1.21 ± 0.14 (mean ± s.e.m., Fisher's *z*-transformed correlation coefficients for the first and penultimate training block, respectively). Thus, practice on the motor sequence resulted not only in faster task execution, but also in the formation of a new, presumably more efficient, pattern for generating the motor sequence.

### Task-Induced Changes in Activity

**Figure 3:**
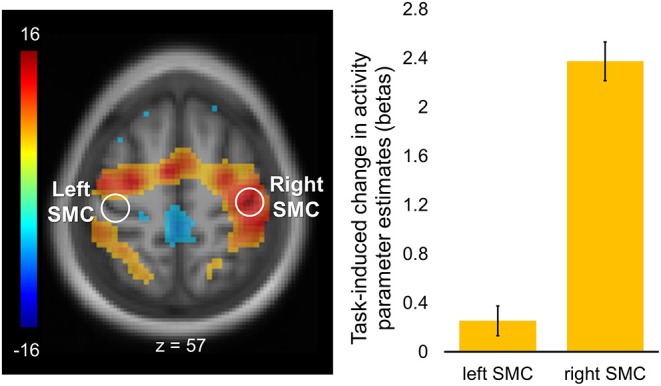
Regions of interest and task-induced changes in activity. A statistical map of the whole-brain analysis of task-induced changes in activity was thresholded at *p* < 0.05 (two-tailed) using peak-level family-wise error (FWE) correction over the entire brain. The map is displayed over the mean structural image of all participants. Color bar represents *t* values with yellow-red and green-blue shades indicating regions with task-induced activity increases and decreases, respectively. Regions of interest (ROIs, white circles) were defined as spheres (*r* = 6 mm) within the hand area of the left (“passive”) and right (“active”) somatomotor cortex (SMC; xyz = −36, −24, 57, and xyz = 42, −21, 57, respectively). Columns represent group mean of task-induced increases in activity for each ROI. Error bars represent standard errors of the mean (s.e.m.).

Task-induced changes in activity are shown in Figure 3. As expected, task-evoked activation was strongly lateralized to the right SMC contralateral to the performing (left) hand with no significant activation of its homolog within the left hemisphere. Cortical activations were also observed within the supplementary motor area (SMA) as well as in the dorsal premotor and parietal regions, bilaterally.

### Task-Induced Changes in Functional Connectivity

**Figure 4:**
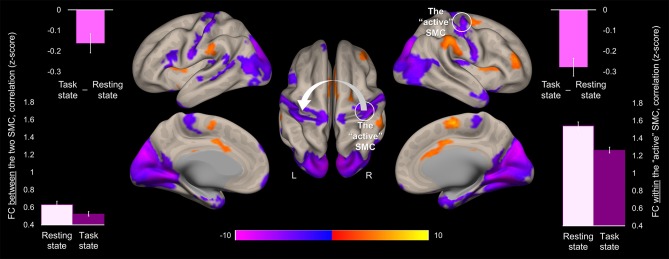
Task-induced change in the whole-brain functional connectivity of the “active” somatomotor cortex. The results of the whole-brain analysis are showing regions where FC with the hand area of the “active” SMC significantly changed during the motor sequence task comparted to the resting state ([Task state]—[Resting state]). The analysis was performed using the ROI within the “active” SMC as a seed (the white circle). The statistical map is displayed over the inflated mean cortical surface of all participants at *p* < 0.001 (two-tailed). A horizontal color bar represents *t* values with red-yellow and blue-magenta shades indicating task-induced stronger and weaker FC with the “active” SMC, respectively. L and R—left and right hemisphere, respectively. The values of the FC between the two SMC (the white arrow) and within the “active” SMC (the white circle) were extracted separately from the resting and task state time-series (lower left and right plot, respectively); the task-induced decreases in the FC strength between the two SMC and within the “active” SMC are also plotted (upper plots). Columns represent group means. Error bars represent standard errors of the mean (s.e.m.). SMC, somatomotor cortex.

Task-induced changes in FC were assessed using a whole-brain functional connectivity analysis approach with the ROI within the “active” SMC as a seed. Comparison of the FC patterns between the resting and task states revealed that during the task the FC strength within the somatomotor network encompassing bilateral somatosensory and motor cortices significantly decreased (Figure 4). These decreases were evident not only in the FC estimates between the two hemispheres but also within the “active” hemisphere contralateral to the performing hand. Specifically, FC values between the two ROIs within the somatomotor hand areas during the task state were significantly lower than during the resting state (Figure 4, left plots), indicating task-induced functional segregation between the two SMC. These decreases reflected only a relative decline in FC between the “active” and “passive” SMC, as indicated by FC values significantly greater than zero during either state (*t* > 17.43, *p* < 0.001). The preserved functional connections between the two SMC during unimanual task are in line with the resilience of intrinsic brain networks to momentary demands of the environment (19). The significant task-induced decreases in the FC strength were also observed within the “active” somatomotor hand area itself, despite the increased task-induced activation of this primary region. Such suppressive effect of the task state on the FC strength within the “active” SMC may derive from selective synchronization and amplification of activity within neural populations that are better suited to elicit the desired action, thereby locally segregating them from other task-irrelevant units within the same ROI. These changes in the FC strength within the “active” SMC were statistically robust, yet, relatively small, in terms of their magnitude, as compared to the particularly high FC values across the two states (mean ± s.e.m: 1.54 ± 0.04 and 1.26 ± 0.03, for the resting and task state, respectively) (Figure 4, right plots), indicating strongly synchronized intrinsic activity between neural populations representing the performing hand. This relative decline in FC strength with the “active” SMC extended beyond the somatomotor hand areas and was widespread along both central sulci. Additional decreases were observed within the occipital lobe, bilaterally. In parallel, the task state resulted in stronger FC between the “active” SMC and higher-level parietal, temporal and prefrontal cortical areas, including the SMA, indicating the need to integrate information from these regions to meet the task goals. Increased FC was also observed within the basal ganglia, including bilateral putamen and thalamus.

### Segregation Within the Somatomotor Network and Learning

We next explored the relationship between FC patterns of the “active” SMC and the effect of learning. To this end, the reduction in block duration when performing the task, as a measure of gains in performance rate, and the degree of tapping pattern similarity by the end of training, as a measure for performance consistency, were calculated for each individual. These measures were entered as covariates of interest in the whole-brain FC analyses using the “active” SMC as a seed (Tables 1, 2).

**Table 1:** Areas where functional connectivity correlated with gains in performance rate.

		**MNI coordinates**			**Peak-level statistics**		**Cluster-level statistics**	
**Label**		**x**	**y**	**z**	***z*-score**	***p***	**# of voxels**	***p***
**1. TASK STATE—RESTING STATE**								
A. Positive correlation								
No areas with significant effect								
B. Negative correlation								
							82	0.002
Frontal_Sup_Medial	L	−6	66	3	4.132	<0.001		
Frontal_Med_Orb	R	6	54	−9	3.632	<0.001		
							33	0.029
Temporal_Pole_Mid	L	−36	9	−45	3.875	<0.001		
Temporal_Pole_Sup	L	−30	15	−33	2.963	0.002		
							57	0.006
Temporal_Mid	L	−60	−15	−18	3.718	<0.001		
Temporal_Inf	L	−57	−21	−24	3.226	<0.001		
**2. RESTING STATE**								
A. Positive correlation								
							40	0.028
Frontal_Med_Orb	L	−9	57	−3	3.831	<0.001		
Frontal_Sup_Medial	L	−6	66	0	3.115	<0.001		
B. Negative correlation								
							84	0.003
SupraMarginal	R	69	−42	27	3.552	<0.001		
Temporal_Sup	R	69	−45	18	2.976	0.002		
**3. TASK STATE**								
A. Positive correlation								
No areas with significant effect								
B. Negative correlation								
Postcentral							13	0.137
*left SMC (xyz = −36, −24, 57)	L	−36	−27	54	3.263	0.046 _FWE*_		

Functional connectivity analyses were performed for the whole-brain using the right sensorimotor cortex as a seed. Gains in speed were included as a covariate of interest. The resulted maps were thresholded at p < 0.005 (two-tailed). The inferences were made at the peak-level using family-wise error correction (FWE) over a small volume of interest. Volumes of interest were defined as spheres (r = 10 mm) around center coordinates of the regions of interest within the hand area of the somatomotor cortices (SMC) and supplementary motor area (SMA) (*). Clusters that survived a cluster-level extent threshold of p < 0.05 are also reported. Cluster labeling was performed using AAL (84).

**Table 2:** Areas where functional connectivity correlated with the tapping pattern consistency.

		**MNI coordinates**			**Peak-level statistics**		**Cluster-level statistics**	
**Label**		**x**	**y**	**z**	***z*-score**	***p***	**# of voxels**	***p***
**1. TASK STATE—RESTING STATE**								
A. Positive correlation								
							56	0.006
Cerebelum_6	R	30	−54	−36	4.005	<0.001		
Cerebelum_Crus1	R	30	−63	−36	3.825	<0.001		
							43	0.015
Frontal_Sup_Medial	R	6	33	57	3.873	<0.001		
B. Negative correlation								
							32	0.031
Precentral	L	−30	−3	48	4.076	<0.001		
**2. RESTING STATE**								
A. Positive correlation								
							112	<0.001
Frontal_Sup_Orb	R	12	72	−3	4.550	<0.001		
Frontal_Med_Orb	L	−3	69	−3	3.859	<0.001		
							65	0.007
Precuneus	L	−12	−60	42	4.110	<0.001		
Parietal_Inf	L	−33	−57	45	3.593	<0.001		
B. Negative correlation								
							121	<0.001
Cerebelum_6	R	30	−54	−36	4.798	<0.001		
Cerebelum_Crus1	R	24	−66	−36	4.327	<0.001		
Cerebelum_8	R	15	−69	−36	3.324	<0.001		
							51	0.015
Cerebelum_Crus1	L	−33	−57	−39	3.394	<0.001		
**3. TASK STATE**								
A. Positive correlation								
							204	<0.001
SupraMarginal	R	57	−45	33	4.274	<0.001		
Angular	R	57	−57	36	3.185	<0.001		
Parietal_Inf	R	57	−42	48	3.894	<0.001		
Temporal_Sup	R	45	−42	3	4.168	<0.001		
							38	0.017
Angular	R	39	−72	42	4.141	<0.001		
							57	0.005
Temporal_Inf	L	−42	9	−39	3.348	<0.001		
							40	0.015
Occipital_Mid	R	30	−90	15	3.459	<0.001		
B. Negative correlation								
Postcentral							18	0.083
*right SMC (xyz = 42, −21, 57)	R	33	−21	57	3.796	0.009_FWE*_		
							44	0.011
Supp_Motor_Area	L	−15	0	54	3.540	<0.001		

Functional connectivity analyses were performed for the whole-brain using the right sensorimotor cortex as a seed. The degree of the tapping pattern consistency was included as a covariate of interest. The resulted maps were thresholded at p < 0.005 (two-tailed). The inferences were made at the peak-level using family-wise error correction (FWE) over a small volume of interest. Volumes of interest were defined as spheres (r = 10 mm) around center coordinates of the regions of interest within the hand area of the somatomotor cortices (SMC)(*). Clusters that survived a cluster-level extent threshold of p < 0.05 are also reported. Cluster labeling was performed using AAL (84).

**Figure 5:**
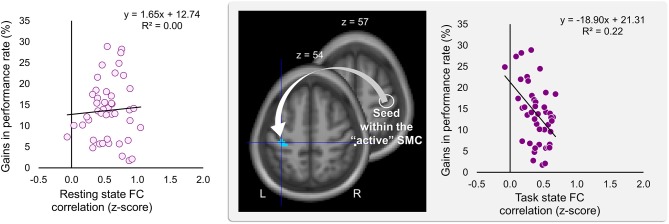
Inverse relationship between individual differences in performance gains and task-based FC strength between the two somatomotor cortices. Gains in performance rate during the training were negatively correlated with the degree of the task-based functional connectivity (FC) between the two somatomotor cortices (SMC). The whole-brain FC analysis was performed on the task state time-series using the ROI within the “active” SMC as a seed (the white circle); individual gains in performance rate were entered as a covariate of interest (middle panel). These gains were calculated as a percentage change in mean performance duration during the last six vs. the first six blocks of training—higher values indicating greater improvement. Gains in performance rate are plotted against FC values extracted from the resting and task state time-series (left and right plot, respectively). The FC values were calculated between the ROI within the “active” SMC and the peak voxel of the significant cluster within the left somatomotor hand area (xyz = −36, −27, 54) (the white arrow). The cyan blob resulted from the statistical maps thresholded at *p* < 0.005 (two-tailed). L and R—left and right hemisphere, respectively.

**Figure 6:**
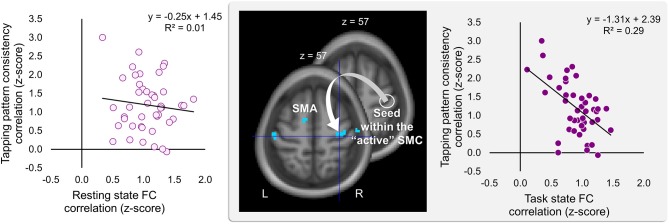
Inverse relationship between individual differences in degree of the tapping pattern consistency attained by the end of training and task-based FC strength within the “active” somatomotor cortex. The degree of consistency in the tapping pattern by the end of training was negatively correlated with the degree of the task-based FC within the hand area of the “active” somatomotor cortex (SMC) as well as between this region and the supplementary motor area (SMA). The whole-brain FC analysis was performed on the task state time-series using the ROI within the “active” SMC as a seed (the white circle); individual measures of the tapping pattern consistency were entered as a covariate of interest (middle panel). The degree of the tapping pattern consistency was assessed by averaging normalized Pearson's correlation coefficients between the tapping patterns generated during the last six blocks excluding block 14 (i.e., blocks 8–13) and the tapping pattern formed by the end of training (i.e., during block 14)—higher values indicating greater tapping pattern similarity and, therefore, greater consistency by the end of training. These coefficients are plotted against FC values extracted from the resting and task state time-series (left and right plot, respectively). The FC values were calculated between the ROI within the “active” SMC and the peak voxel of the significant cluster within the somatomotor hand area (xyz = 33, −21, 57) (the white arrow). The cyan blobs resulted from the statistical maps thresholded at *p* < 0.005 (two-tailed). L and R—left and right hemisphere, respectively.

Significant effects within the somatomotor network were observed only when the correlation analyses were performed on the task-based FC maps; no significant correlation was evident with FC estimates within the somatomotor network during the resting state either with their relative changes when comparing between the two states (brain regions that exhibited significant effect are listed in Table 1). Specifically, during the task, individual differences of gains in performance rate were associated with weaker FC of the seed ROI (the “active” SMC) with its homolog in the left hemisphere (Figure 5, right panel; Table 1.3). Importantly, despite the fact that the analysis was conducted across the entire brain, the significant effect was notable only around the hand knob. This result may indicate that reduced influences of somatomotor representations of the passive hand on ongoing activity of somatomotor representations of the active hand facilitated the development of faster performance rate. Individual differences in the degree of tapping pattern consistency, on the other hand, were associated with weaker FC within the “active” somatomotor hand area itself (Figure 6, right panel; Table 2.3). A similar association was found with FC values between the seed ROI and SMA. These results link more consistent performance by the end of training with segregation processes within the “active” SMC and, presumably, its selective information integration with the SMA. Note that all associations were negative (i.e., individuals with greater gains in performance rate and greater consistency by the end of training had reduced seed-based FC within the somatomotor network) and specifically present during the task state (see Table 3 for detailed statistics of correlation analyses) but not during the resting state (see also left graphs in Figures 5, 6). The direct comparison between correlations resulted in significant effect of state (*z* > 2.53, *p* < 0.01), confirming that the relationship between FC values and learning measures differed between the resting and task state. The significant difference between the two states suggests that FC patterns within the somatomotor network were reconfigured during the task. Only these reconfigured patterns predicted individual differences in learning.

**Table 3:** Correlations between the FC strength and behavioral correlates of learning.

	**Resting state**		**Task state**		**Correlations' comparison between the states**	
	***r***	***p***	***r***	***p***	***z***	***p***
Gains in performance rate in association with:						
FC with the “passive” SMC	0.58	0.71	−0.47^**^	0.001	2.53^*^	0.006
Tapping pattern consistency in association with:						
FC within the “active” SMC	−0.11	0.47	−0.54^**^	<0.001	2.56^*^	0.005
FC with the SMA	−0.003	0.99	−0.51^**^	<0.001	2.69^*^	0.004

* Significant results at 0.01 level;

** *Significant results at 0.001 level*.

**Figure 7:**
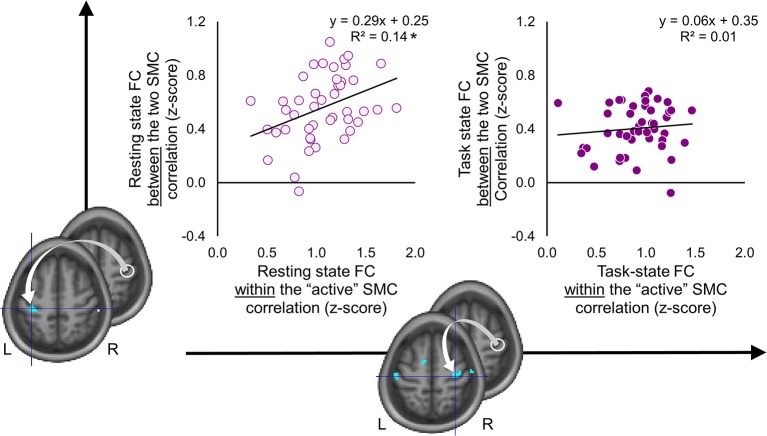
Task-induced segregation between the two somatomotor cortices. Individual functional connectivity (FC) values between the two somatomotor cortices (SMC) (vertical axis) are plotted against FC values within the “active” SMC (horizontal axis) during the resting and task state (left and right plots, respectively). The FC values were calculated between the ROI within the “active” SMC (white circle) and peak voxels of clusters resulted from the whole-brain FC analyses, which were conducted using behavioral measures as covariates of interest (for details see Figures 5, 6). L and R—left and right hemisphere, respectively. ^*^significant correlation at 0.01 level. During the resting state, FC between the two SMC was positively correlated with FC within the “active” SMC. No such relationship was observed during the task state.

As the results reported above suggest, the two SMC were functionally segregated during the task as compared to the resting state (Figure 4). The task-induced segregation, as reflected in the reduced FC strength, was also evident within the “active” SMC itself. Did the suppressive effect of the task state on the FC strength extend to the somatomotor representations linked to learning? If so, does such effect indicate functional segregation or merely an overall reduction of noise correlations—a phenomenon suggested by animal studies that may lead to a widespread reduction in connectivity strength at the level of neural populations (54)? To answer these questions, we performed correlation analyses on the individual FC values extracted from clusters (peak voxels) where decreased FC with the seed (i.e., the ROI within the “active” SMC) was associated with learning; these clusters are shown in Figures 5, 6. During the resting state, participants who showed stronger FC within the “active” SMC itself, also showed stronger FC between the two SMC (*r* = 0.38, *p* = 0.01) (Figure 7, left plot). However, no significant correlation between these estimates of intra- and inter-hemispheric interactions was observed during the task state (*r* = 0.10, *p* = 0.51) (Figure 7, right plot). This finding, which indicates that relationship between the intra- and inter-hemispheric interactions differed between the two states, rules out the possibility that suppressive effect of the task state on the FC strength within the somatomotor network can be fully explained by the overall noise reduction. Instead, it suggests that ongoing activity within the “active” and “passive” SMC became less synchronized during the task compared to the resting state and thereby indicates task-induced segregation. This segregation was specifically present between somatomotor representations in each hemisphere linked with different aspects of learning.

The same analysis performed on the individual FC values of the seed ROI with the peak of the significant clusters within the “active” SMC and SMA showed an inverse relationship. No significant correlation between these values was observed during the resting state (*r* = 0.18, *p* = 0.25). However, during the task state, participants with stronger FC within the “active” SMC itself also showed stronger FC between the seed ROI and SMA (*r* = 0.45, *p* < 0.01). This finding indicates that ongoing activity of the specific neural populations within the “active” SMC and SMA, whose FC patterns during the task were inversely related to more consistent performance by the end of training, was also stronger synchronized during the task but not during the resting state.

### Differences in Performance Rate as a Possible Confound

The overall time spent on the actual task performance varied between participants as a function of their performance rate so that faster performers spent less time on the task than slower performers. Therefore, it is possible that the current pattern of results might be confounded by the inter-individual variation in the length of the time series used to estimate the task-based FC. However, neither the gains in performance rate nor the degree of the tapping pattern consistency by the end of training were significantly correlated with the individuals' time spent on the actual task performance (|*r*| < 0.20, *p* > 0.18). Therefore, the possibility that the current pattern of results can be fully explained by the differences in the overall performance rate or the length of the time series is unlikely.

## Discussion

In the current study, we investigated how functional connectivity within the somatomotor network is reconfigured during a unimanual motor sequence task, and how these changes are related to individual learning capacities. To do so, we applied seed-driven functional connectivity analysis to fMRI data collected in a previous study (43). Participants were scanned during resting state, as well as during a motor sequence task, which required them to repeatedly generate a five-element sequence using their non-dominant hand. Our results suggest that unimanual performance of the motor sequence relies on functional segregation between the two SMC and selective integration between the SMC engaged in the task and the SMA. We thus provide supportive evidence to the notion that task-induced changes in FC reflect reconfiguration of the connectivity patterns within the somatomotor network rather than a simple amplification or silencing of its intrinsic dynamics. Such reconfiguration, as captured with the BOLD-fMRI signal, not only support motor behavior but may also predict learning capacity.

### The Widespread Task-Induced Reduction in Functional Connectivity

Here we show that the unimanual motor sequence task induced significant reduction in the FC of the “active” SMC with extensive regions along the central sulcus, bilaterally. Such suppressive effect of the task state on the FC strength is consistent with previous studies that also compared FC patterns between the resting and task states and reported task-induced FC decreases within the somatomotor network (13, 28, 85). However, these within-network decreases were observed across various paradigms, including passive movie watching (28), thereby raising the possibility that FC suppression within the somatomotor network characterizes transitions between brain states (i.e., from the resting to task state) but may not reflect specific processes related to motor action. Moreover, previous findings suggest that the suppressive effect of the various tasks on the FC strength extends beyond the somatomotor network and may be a core feature of local brain circuits regardless of their network affiliation, functional properties or cognitive demands of the task (31, 86, 87).

Indeed, the widespread task-induced reduction in FC strength reported by fMRI studies may reflect a global suppression of noise correlations. Such interpretation is in line with the results from animal research that has shown attenuation in correlated variability of neurons' firing rate, which is commonly considered as noise, upon various stimuli and task events (54, 55, 88). This effect was observed across different neural populations regardless of their tuning properties lending support to the notion that the suppression of neural variability may be an overall feature of the cortical response to the task state (54). At the network level, such reduction of noise implies that functional circuits become more stable when driven by stimulus or task. Nevertheless, the reduction of correlated noise lacks specificity and, by itself, does not improve fidelity of neural encoding (55, 56). If, however, the noise reduction is correlated with the task-relevant signal, it could improve encoding accuracy and facilitate learning (89, 90).

### Reduction in Functional Connectivity and Selective Engagement of Task-Relevant Neural Representations

As predicted in the current study, the task-evoked activation within the somatomotor hand area was lateralized to the hemisphere contralateral to the performing hand [e.g., (2, 91–93)], in line with the known phenomenon of lateralization of somatomotor representations specifically tuned to movements generated by contralateral body parts (94). Given the well-defined somatotopic organization of these representations along the central sulcus, here we argue that the widespread and local task-induced reduction in FC with the “active” SMC may reflect different neurophysiological processes. Specifically, the suppressive effect of the task state on FC strength between the two SMC may derive from the overall non-selective suppression of spontaneous activity to reduce noise correlations. Such noise reduction may also explain task-induced decreases in FC between the seed ROI within the “active” SMC and somatomotor representations of other body parts. The FC suppression within the “active” somatomotor hand area itself, however, may further reflect selective co-activation of neural populations that are particularly tuned to perform the finger tapping task, thereby segregating them from other intrinsically connected, but task-irrelevant units. Supporting fMRI evidence of non-selective decreases in local FC driven by a finger tapping task has been provided by Lv et al. (95), who showed that both fast and slow finger tapping rates have a similar suppressive effect on local FC in the SMC ipsilateral (“passive”) to the performing hand. The effect observed within the contralateral (“active”) SMC, however, was different, such that the faster tapping rate, which usually results in stronger activation of this region (96–98), was associated with greater reduction in its local FC, hence indicating a greater segregation. Thus, the combination of increased task demands, the stronger activation and the more segregated activity within the contralateral SMC support the idea that FC dynamics within the “active” somatomotor hand area observed in our study constitute a signature for selective engagement of task-relevant neural representations during motor task execution.

The idea of selective engagement of task-relevant neural representations resonates with the emerging recognition that task-induced changes in FC reflect rapid reconfiguration of functional connections (26, 27, 42). Evidently, these changes are relatively small, in terms of their magnitude, as they are probably constrained by mainly invariant large-scale functional brain network topography (13, 14). Nevertheless, some characteristics of such changes are specific to the task at hand, allowing to accurately decode the task state of a participant (29, 30), and are linked to better performance (27, 31–38). Notably, weaker correspondence between FC patterns during the resting and task states particularly characterizes primary sensory and motor circuits (42). Such deviation from the intrinsic brain dynamics may depend on attentional state, stimulus properties, and task complexity (27, 35, 42, 99), hence supporting the idea that transient sub-networks within the sensory and motor circuits are formed to process incoming information or carry out the desired action. Here we show that reconfigured FC patterns within the somatomotor network are not only behaviorally relevant, but may also support learning. In fact, better learning, which was expressed as a faster and more consistent performance by the end of training, was related to individual differences in the FC strength during the task, but not during the resting state. Such dissociation is consistent with the “idling” view on the intrinsic brain function during the resting state (26) and suggests that reconfigured FC patterns within the somatomotor network during the task can capture the neural dynamics that sub-serve learning.

### Task-Based Functional Connectivity Strength Is Inversely Related to Learning

Behaviorally, the beneficial effects of motor sequence practice were assessed based on improved performance rate, which can be achieved by simple acceleration of single movements reflecting learning at the executive level, and greater tapping pattern consistency, which presumably reflects formation of internal sequence representation (100, 101). Here we show that these two complementary metrics to estimate learning are differentially associated with individual differences in FC strength between the two SMC and within the “active” SMC itself.

Particularly, participants who exhibited weaker FC between the two SMC showed greater improvement in performance rate. The effect within the “passive” SMC was localized to the hand knob, indicating that greater inter-hemispheric segregation between somatomotor units representing hand movements facilitated learning at the executive level. Such increased autonomy between the two somatomotor hand areas may reflect the release from inter-hemispheric inhibition—an effect postulated by transcranial magnetic stimulation (TMS) studies (102, 103). In fact, it has been shown that virtual “lesion” to the “passive” SMC induced by repetitive TMS leads to improved performance in the ipsilateral hand, presumably due to the suppressed inter-hemispheric inhibition (104–108). The inter-hemispheric inhibition is also reduced following unimanual training (109–111) and is associated with faster performance not only in the trained, but in the untrained hand as well (111). Alternatively, the improved performance associated with greater segregation between the two SMC reported here may also indicate beneficial effects of release from irrelevant somatosensory input from the “passive” hemisphere. Indeed, the cluster showing significant effect within the “passive” SMC is located slightly posteriorly to the central sulcus encompassing the somatosensory hand area. To reliably dissociate between somatosensory and motor representations nested closely together around the hand knob, however, higher spatial resolution of the fMRI data is required. In any case, we suggest that the facilitatory effect of greater segregation between somatomotor representations is selective to the effector engaged in the task (i.e., the hand as compared to other body parts). Thus, effector-selective “pruning” of inter-hemispheric connections may facilitate learning primarily at the level of motor execution rather than movement synergy and sequence representation. It is worth noting, however, that under certain conditions, influences from the “passive” SMC are excitatory and may facilitate performance in a sequence-specific manner (48, 112).

In addition to a faster performance rate, which may develop due to more efficient execution of single movements regardless of their serial order, repeated experience with the motor sequence also shapes the tapping pattern of that sequence (76). Being determined by the relative spacing between the keypresses, this pattern may vary at the beginning, but stabilizes by the end of training (75). In the current study, participants who expressed a more stable performance by the end of training, hence generating a highly reproducible pattern when tapping the sequence, also exhibited weaker FC within the “active” SMC itself during the task. Animal studies suggest that same movements can be generated by various activity patterns within the motor cortex (64, 113). With repeated experience, however, the variability in these patterns decreases (64, 114). The greater reproducibility of the spatiotemporal patterns of neural activity concurs with the emergence of movement stereotypy and, therefore, may indicate the formation of dedicated internal representations of a new motor synergy. During initial phases of skill acquisition, these neural representations are shaped through selective activation of specialized neural populations and tuning of their firing rate (115). Evidence from fMRI studies on motor sequence learning in humans points out to similar processes (46, 47, 52). Our current results are thus consistent with the existence of an experience-driven mechanism of selective tuning and stabilization among neural populations representing the performing hand. We suggest that greater selectivity and stabilization of task-relevant representations are reflected in the reduced FC strength within the “active” SMC during the task.

Concurrently, the degree of task-based FC strength between the “active” SMC and SMA was also inversely related to the degree of performance consistency. Together with task-induced integration between these two regions, which was indicated by increases in FC strength during the task compared to the resting state, such relationship suggests that selective tuning at the lower level of primary somatomotor cortex may be governed by higher level processes within the SMA. The latter interpretation relies on three widely accepted views that are strongly supported by animal and human studies. First, the SMA has direct projections to the primary motor cortex (116). These projections are primarily excitatory (117) and are organized bilaterally with no clear lateralization (118). Second, the SMA is situated high within the hierarchy of the motor control system and is involved in initiation, monitoring and regulation of voluntary movements (119–122). Finally, this supra-motor region is crucially involved in sequencing of actions (123–125) and representations of practiced motor sequences (126–129). Accordingly, our results add up to accumulative fMRI evidence suggesting that SMA plays a role in encoding a sequence-specific pattern of finger movements (130) and orchestrates processes of rapid reorganization within the “active” SMC.

### Methodological Considerations

Currently, there is a growing interest to study cognitive brain function using large-scale network modeling [for the recent reviews, please see (131, 132)]. This approach has been developed upon the foundations of graph theory by leveraging the mathematical description of a graph, which is composed of nodes and weighted edges, to represent brain networks. Nodes are commonly chosen as contiguous volumes/regions with boundaries defined either anatomically, using parcellation atlases, or functionally, using community detection algorithms. Edge weights are commonly defined by a degree of correlation or coherence between pairs of nodes. The sensitivity of this approach to dynamic changes within and between functional brain circuits depends on the size of each node, which consequently determines their overall number, and the way these nodes are grouped into networks. Whereas, both factors alleviate the multiple comparison problem, since they reduce high dimensionality of the whole-brain fMRI data, as a drawback, they also inherently reduce local specificity, thereby limiting special resolution of investigations to large-scale changes. For example, testing the integration of large-scale functional neural circuitry during the unimanual motor task, when participants practiced to generate different sequences upon visual guidance, Bassett et al. (41) provided evidence for growing autonomy between the visual and somatomotor systems over the course of a 6-week training (41). This segregation was also paralleled by disengagement of cognitive control networks including connections originated from frontal and anterior cingulate cortices. Whereas, such autonomy is consistent with the obvious decreased dependency on the visual cue and higher cognitive processes with practice [see also (36)], the researchers reported no significant changes in the overall degree of integration/segregation within the somatomotor network, which included primary somatosensory and motor regions representing both the active and passive hands, as well as SMA. However, when the analysis was conducted using the intra-network integration values calculated for each area separately, it revealed significant within-network changes that were associated with the amount of training [Supplementary Figure 8 in Bassett et al. (41)]. The results of the current study are consistent with this supplementary finding and further suggest that experience-driven reorganization within the somatomotor network occurs early in learning and involves both segregation and integration.

There are several key advantages of our study as compared to others in the literature. First, it is worth noting that our experimental design employed a motor sequence paradigm without any external input or feedback during continuous periods of actual performance, thereby, minimizing influences of complex interactions among heterogeneous networks during the task state. The only external cue provided during the training was a change in color of the fixation cross, from red to green and from green to red, that indicated the beginning and the end of each training block, respectively. Second, this design also allowed us to separate the functional data collected during continuous performance periods (i.e., performance blocks) from the interleaved periods of rest. Finally, continuous nature of actual performance upon self-guided regime was advantageous for separation of FC measures from activation biases evoked at task transitions. In that way, FC values were calculated based on ongoing fluctuations of the fMRI signal during continuous periods of either resting or task state, thereby providing comparable estimates of connectivity patterns between those states.

## Conclusion

Our results suggest that a hypothesis-driven seed-based FC approach reliably captures the task-induced changes in functional connectivity within the somatomotor network during the unimanual motor sequence task. These changes are not limited to the task-activated regions, hence revealing the existence of distributed processes of segregation and integration that act in parallel to allow for the generation of fine motor movements. These reconfigured connectivity patterns not only support task execution but also facilitate learning. The limited correspondence between the patterns of task-induced activity and connectivity is a known phenomenon within the scientific community. While there is an increasing recognition of potential benefits of combining the assessment of both metrics obtained from the task-based fMRI data series in order to understand cognitive brain functions (133), here we show that a careful consideration of the activation profile and functional specialization of each region of interest is not only desirable, but also critical to draw meaningful conclusions based on FC measures. In the current study, this approach, in combination with reliable behavioral correlates of motor sequence learning, allowed us to tease apart neural dynamics that may drive the adaptive processes during the initial phases of skill acquisition, the ones that need to be “silenced” and the ones that should be selectively pruned to rule out their task-irrelevant influences. Our results suggest that during the task, more segregated activity patterns between neural populations representing hand movements within the somatomotor cortex were beneficial for the development of both faster and more consistent performance by the end of training. Whereas, greater segregation between the two SMC may indicate effector-specific pruning of inter-hemispheric connections, which may facilitate gains at the level of motor execution, the same effect observed within the “active” SMC may possibly indicate the existence of selective tuning and stabilization processes, thereby resulting in more reproducible patterns of activity that allow to generate the motor sequence with greater consistency.

## Data Availability Statement

The datasets generated for this study will not be made publicly available because participants did not provide their explicit consent for public data sharing. Requests to access the datasets should be directed to JD, julien.doyon@mcgill.ca.

## Ethics Statement

The studies involving human participants were reviewed and approved by the Research ethics board of the RNQ (Regroupement Neuroimagerie Québec). The patients/participants provided their written informed consent to participate in this study.

## Author Contributions

GA and JD designed the experiment. GA performed the experiment. EG analyzed the data. EG and JD wrote the original draft. GA and OL reviewed and edited the manuscript. OL provided expertise and feedback.

### Conflict of Interest

The authors declare that the research was conducted in the absence of any commercial or financial relationships that could be construed as a potential conflict of interest.

## References

[1] Belliveau JW Kennedy DN McKinstry RC Buchbinder BR Weisskoff RM Cohen MS. Functional mapping of the human visual cortex by magnetic resonance imaging. Science. (1991) 254:716–9. 10.1126/science.1948051 1948051

[2] Kwong KK Belliveau JW Chesler DA Goldberg IE Weisskoff RM Poncelet BP. Dynamic magnetic resonance imaging of human brain activity during primary sensory stimulation. Proc Natl Acad Sci USA. (1992) 89:5675–9. 10.1073/pnas.89.12.5675 1608978 PMC49355

[3] Davis T Poldrack RA. Measuring neural representations with fMRI: practices and pitfalls. Ann N Y Acad Sci. (2013) 1296:108–34. 10.1111/nyas.12156 23738883

[4] Poldrack RA. Imaging brain plasticity: conceptual and methodological issues - a theoretical review. Neuroimage. (2000) 12:1–13. 10.1006/nimg.2000.0596 10875897

[5] Friston KJ Frith CD Liddle PF Frackowiak RSJ. Functional connectivity: the principal-component analysis of large (PET) data sets. J Cereb Blood Flow Metab. (1993) 13:5–14. 10.1038/jcbfm.1993.4 8417010

[6] Tyszka JM Kennedy DP Adolphs R Paul LK. Intact bilateral resting-state networks in the absence of the corpus callosum. J Neurosci. (2011) 31:15154–62. 10.1523/JNEUROSCI.1453-11.2011 22016549 PMC3221732

[7] Lowe MJ Mock BJ Sorenson JA. Functional connectivity in single and multislice echoplanar imaging. Neuroimage. (1998) 7:119–32. 10.1006/nimg.1997.0315 9558644

[8] He BJ Snyder AZ Zempel JM Smyth MD Raichle ME. Electrophysiological correlates of the brain's intrinsic large-scale functional architecture. Proc Natl Acad Sci USA. (2008) 105:16039–44. 10.1073/pnas.0807010105 18843113 PMC2564983

[9] Power JD Cohen AL Nelson SM Wig GS Barnes KA Church JA. Functional network organization of the human brain. Neuron. (2011) 72:665–78. 10.1016/j.neuron.2011.09.006 22099467 PMC3222858

[10] Yeo BTT Krienen FM Sepulcre J Sabuncu MR Lashkari D Hollinshead M. The organization of the human cerebral cortex estimated by intrinsic functional connectivity. J Neurophysiol. (2011) 106:1125–65. 10.1152/jn.00338.2011 21653723 PMC3174820

[11] Biswal B Yetkin FZ Haughton VM Hyde JS. Functional connectivity in the motor cortex of resting human brain using echo-planar MRI. Magn Reson Med. (1995) 34:537–41. 10.1002/mrm.1910340409 8524021

[12] Zhu Y Cheng L He N Yang Y Ling H Ayaz H. Comparison of functional connectivity estimated from concatenated task-state data from block-design paradigm with that of continuous task. Comput Math Methods Med. (2017) 2017:1–11. 10.1155/2017/4198430 28191030 PMC5278200

[13] Gratton C Laumann TO Gordon EM Adeyemo B Petersen SE. Evidence for two independent factors that modify brain networks to meet task goals. Cell Rep. (2016) 17:1276–88. 10.1016/j.celrep.2016.10.002 27783943 PMC5123792

[14] Cole MW Bassett DS Power JD Braver TS Petersen SE. Intrinsic and task-evoked network architectures of the human brain. Neuron. (2014) 83:238–51. 10.1016/j.neuron.2014.05.014 24991964 PMC4082806

[15] Smith SM Fox PT Miller KL Glahn DC Fox PM Mackay CE. Correspondence of the brain's functional architecture during activation and rest. Proc Natl Acad Sci USA. (2009) 106:13040–5. 10.1073/pnas.0905267106 19620724 PMC2722273

[16] Fox MD Snyder AZ Zacks JM Raichle ME. Coherent spontaneous activity accounts for trial-to-trial variability in human evoked brain responses. Nat Neurosci. (2006) 9:23–5. 10.1038/nn1616 16341210

[17] Fox MD Snyder AZ Vincent JL Raichle ME. Intrinsic fluctuations within cortical systems account for intertrial variability in human behavior. Neuron. (2007) 56:171–84. 10.1016/j.neuron.2007.08.023 17920023

[18] Mennes M Kelly C Zuo XN Di Martino A Biswal BB Castellanos FX. Inter-individual differences in resting-state functional connectivity predict task-induced BOLD activity. Neuroimage. (2010) 50:1690–701. 10.1016/j.neuroimage.2010.01.002 20079856 PMC2839004

[19] Raichle ME. Two views of brain function. Trends Cogn Sci. (2010) 14:180–90. 10.1016/j.tics.2010.01.008 20206576

[20] Deco G Jirsa VK McIntosh AR. Emerging concepts for the dynamical organization of resting-state activity in the brain. Nat Rev Neurosci. (2011) 12:43–56. 10.1038/nrn2961 21170073

[21] Tavor I Parker Jones O Mars RB Smith SM Behrens TE Jbabdi S. Task-free MRI predicts individual differences in brain activity during task performance. Science. (2016) 352:216–20. 10.1126/science.aad8127 27124457 PMC6309730

[22] Ito T Kulkarni KR Schultz DH Mill RD Chen RH Solomyak LI. Cognitive task information is transferred between brain regions via resting-state network topology. Nat Commun. (2017) 8:1–13. 10.1038/s41467-017-01000-w 29044112 PMC5715061

[23] Mattar MG Wymbs NF Bock AS Aguirre GK Grafton ST Bassett DS. Predicting future learning from baseline network architecture. Neuroimage. (2018) 172:107–17. 10.1016/j.neuroimage.2018.01.037 29366697 PMC5910215

[24] King BR Van Ruitenbeek P Leunissen I Cuypers K Heise K-F Santos Monteiro T. Age-related declines in motor performance are associated with decreased segregation of large-scale resting state brain networks. Cereb Cortex. (2018) 28:4390–402. 10.1093/cercor/bhx297 29136114 PMC6215458

[25] Cole MW Ito T Bassett DS Schultz DH. Activity flow over resting-state networks shapes cognitive task activations. Nat Neurosci. (2016) 19:1718–26. 10.1038/nn.4406 27723746 PMC5127712

[26] Betti V DellaPenna S de Pasquale F Mantini D Marzetti L Romani GL. Natural scenes viewing alters the dynamics of functional connectivity in the human brain. Neuron. (2013) 79:782–97. 10.1016/j.neuron.2013.06.022 23891400 PMC3893318

[27] Spadone S Della Penna S Sestieri C Betti V Tosoni A Perrucci MG. Dynamic reorganization of human resting-state networks during visuospatial attention. Proc Natl Acad Sci USA. (2015) 112:8112–7. 10.1073/pnas.1415439112 26080395 PMC4491799

[28] Lynch LK Lu KH Wen H Zhang Y Saykin AJ Liu Z Task-evoked functional connectivity does not explain functional connectivity differences between rest and task conditions. Hum Brain Mapp. (2018) 39:4939–48. 10.1002/hbm.24335 30144210 PMC6397020

[29] Gonzalez-Castillo J Hoy CW Handwerker DA Robinson ME Buchanan LC Saad ZS. Tracking ongoing cognition in individuals using brief, whole-brain functional connectivity patterns. Proc Natl Acad Sci USA. (2015) 112:8762–7. 10.1073/pnas.1501242112 26124112 PMC4507216

[30] Shirer WR Ryali S Rykhlevskaia E Menon V Greicius MD. Decoding subject-driven cognitive states with whole-brain connectivity patterns. Cereb Cortex. (2012) 22:158–65. 10.1093/cercor/bhr099 21616982 PMC3236795

[31] Cohen JR D'Esposito M. The segregation and integration of distinct brain networks and their relationship to cognition. J Neurosci. (2016) 36:12083–94. 10.1523/JNEUROSCI.2965-15.2016 27903719 PMC5148214

[32] Hearne LJ Cocchi L Zalesky A Mattingley JB. Reconfiguration of brain network architectures between resting-state and complexity-dependent cognitive reasoning. J Neurosci. (2017) 37:8399–411. 10.1523/JNEUROSCI.0485-17.2017 28760864 PMC6596866

[33] Hasson U Nusbaum HC Small SL. Task-dependent organization of brain regions active during rest. Proc Natl Acad Sci USA. (2009) 106:10841–6. 10.1073/pnas.0903253106 19541656 PMC2705532

[34] Alavash M Tune S Obleser J. Modular reconfiguration of an auditory control brain network supports adaptive listening behavior. Proc Natl Acad Sci USA. (2018) 116:660–9. 10.1073/pnas.1815321116 30587584 PMC6329957

[35] Demirtaş M Ponce-Alvarez A Gilson M Hagmann P Mantini D Betti V. Distinct modes of functional connectivity induced by movie-watching. Neuroimage. (2019) 184:335–48. 10.1016/j.neuroimage.2018.09.042 30237036 PMC6248881

[36] Mohr H Wolfensteller U Betzel RF Mišić B Sporns O Richiardi J. Integration and segregation of large-scale brain networks during short-term task automatization. Nat Commun. (2016) 7:13217. 10.1038/ncomms13217 27808095 PMC5097148

[37] Braun U Schäfer A Walter H Erk S Romanczuk-Seiferth N Haddad L. Dynamic reconfiguration of frontal brain networks during executive cognition in humans. Proc Natl Acad Sci USA. (2015) 112:11678–83. 10.1073/pnas.1422487112 26324898 PMC4577153

[38] Shine JM Bissett PG Bell PT Koyejo O Balsters JH Gorgolewski KJ. The dynamics of functional brain networks: integrated network states during cognitive task performance. Neuron. (2016) 92:544–54. 10.1016/j.neuron.2016.09.018 27693256 PMC5073034

[39] King DR de Chastelaine M Elward RL Wang TH Rugg MD. Recollection-related iIncreases in functional connectivity predict individual differences in memory accuracy. J Neurosci. (2015) 35:1763–72. 10.1523/JNEUROSCI.3219-14.2015 25632149 PMC4308612

[40] Cohen JR Gallen CL Jacobs EG Lee TG D'Esposito M. Quantifying the reconfiguration of intrinsic networks during working memory. PLoS ONE. (2014) 9:e106636. 10.1371/journal.pone.0106636 25191704 PMC4156328

[41] Bassett DS Yang M Wymbs NF Grafton ST. Learning-induced autonomy of sensorimotor systems. Nat Neurosci. (2015) 18:744–51. 10.1038/nn.3993 25849989 PMC6368853

[42] Mennes M Kelly C Colcombe S Xavier Castellanos F Milham MP The extrinsic and intrinsic functional architectures of the human brain are not equivalent. Cereb Cortex. (2013) 23:223–9. 10.1093/cercor/bhs010 22298730 PMC3513960

[43] Albouy G Fogel SM King BR Laventure S Benali H Karni A. Maintaining vs. enhancing motor sequence memories: respective roles of striatal and hippocampal systems. Neuroimage. (2015) 108:423–34. 10.1016/j.neuroimage.2014.12.049 25542533

[44] Miller GA The magical number seven, plus or minus two: some limits on our capacity for processing information. Psychol Rev. (1956) 63:81–97. 10.1037/h0043158 13310704

[45] Bassett DS Wymbs NF Porter MA Mucha PJ Carlson JM Grafton ST. Dynamic reconfiguration of human brain networks during learning. Proc Natl Acad Sci USA. (2011) 108:7641–6. 10.1073/pnas.1018985108 21502525 PMC3088578

[46] Karni A Meyer G Jezzard P Adams MM Turner R Ungerleider LG. Functional MRI evidence for adult motor cortex plasticity during motor skill learning. Nature. (1995) 377:155–8. 10.1038/377155a0 7675082

[47] Gabitov E Manor D Karni A. Patterns of modulation in the activity and connectivity of motor cortex during the repeated generation of movement sequences. J Cogn Neurosci. (2015) 27:736–51. 10.1162/jocn_a_00751 25390206

[48] Gabitov E Manor D Karni A. Learning from the other limb's experience: sharing the “trained” M1's representation of the motor sequence knowledge. J Physiol. (2016) 594:169–88. 10.1113/JP270184 26442464 PMC4704505

[49] Cole MW Ito T Schultz D Mill R Chen R Cocuzza C. Task activations produce spurious but systematic inflation of task functional connectivity estimates. Neuroimage. (2019) 189:1–18. 10.1016/j.neuroimage.2018.12.054 30597260 PMC6422749

[50] Vines BW Cerruti C Schlaug G. Dual-hemisphere tDCS facilitates greater improvements for healthy subjects' non-dominant hand compared to uni-hemisphere stimulation. BMC Neurosci. (2008) 9:103: 10.1186/1471-2202-9-103 18957075 PMC2584652

[51] Duque J Mazzocchio R Stefan K Hummel F Olivier E Cohen LG. Memory formation in the motor cortex ipsilateral to a training hand. Cereb Cortex. (2008) 18:1395–406. 10.1093/cercor/bhm173 17928331

[52] Gabitov E Manor D Karni A. Done that: short-term repetition related modulations of motor cortex activity as a stable signature for overnight motor memory consolidation. J Cogn Neurosci. (2014) 26:2716–34. 10.1162/jocn_a_00675 24893741

[53] Yokoi A Arbuckle SA Diedrichsen J. The role of human primary motor cortex in the production of skilled finger sequences. J Neurosci. (2018) 38:2798–17. 10.1523/JNEUROSCI.2798-17.2017 29305534 PMC6705846

[54] Churchland MM Yu BM Cunningham JP Sugrue LP Cohen MR Corrado GS. Stimulus onset quenches neural variability: a widespread cortical phenomenon. Nat Neurosci. (2010) 13:369–78. 10.1038/nn.2501 20173745 PMC2828350

[55] Ecker A Berens P Tolias AS Bethge M. The effect of noise correlations in populations of diversely tuned neurons. J Neurosci. (2011) 31:14272–83. 10.1523/JNEUROSCI.2539-11.2011 21976512 PMC3221941

[56] Gu Y Liu S Fetsch CR Yang Y Fok S Sunkara A Angelaki DE. Perceptual learning reduces interneuronal correlations in macaque visual cortex. Neuron. (2011) 71:750–61. 10.1016/j.neuron.2011.06.015 21867889 PMC3163063

[57] Cona G Semenza C. Supplementary motor area as key structure for domain-general sequence processing: a unified account. Neurosci Biobehav Rev. (2017) 72:28–42. 10.1016/j.neubiorev.2016.10.033 27856331

[58] Zeharia N Hertz U Flash T Amedi A. Negative blood oxygenation level dependent homunculus and somatotopic information in primary motor cortex and supplementary motor area. Proc Natl Acad Sci USA. (2012) 109:18565–70. 10.1073/pnas.1119125109 23086164 PMC3494917

[59] Fried I Katz A McCarthy G Sass KJ Williamson P Spencer SS. Functional organization of human supplementary motor cortex studied by electrical stimulation. J Neurosci. (1991) 11:3656–66. 10.1523/JNEUROSCI.11-11-03656.1991 1941101 PMC6575551

[60] Rissman J Gazzaley A D'Esposito M. Measuring functional connectivity during distinct stages of a cognitive task. Neuroimage. (2004) 23:752–63. 10.1016/j.neuroimage.2004.06.035 15488425

[61] Kim JH Lee JM Jo HJ Kim SH Lee JH Kim ST. Defining functional SMA and pre-SMA subregions in human MFC using resting state fMRI: functional connectivity-based parcellation method. Neuroimage. (2010) 49:2375–86. 10.1016/j.neuroimage.2009.10.016 19837176 PMC2819173

[62] Isomura Y Harukuni R Takekawa T Aizawa H Fukai T. Microcircuitry coordination of cortical motor information in self-initiation of voluntary movements. Nat Neurosci. (2009) 12:1586–93. 10.1038/nn.2431 19898469

[63] Churchland MM Cunningham JP Kaufman MT Foster JD Nuyujukian P Ryu SI. Neural population dynamics during reaching. Nature. (2012) 487:51–6. 10.1038/nature11129 22722855 PMC3393826

[64] Peters AJ Chen SX Komiyama T. Emergence of reproducible spatiotemporal activity during motor learning. Nature. (2014) 510:263–7. 10.1038/nature13235 24805237

[65] Korman M Raz N Flash T Karni A. Multiple shifts in the representation of a motor sequence during the acquisition of skilled performance. Proc Natl Acad Sci USA. (2003) 100:12492–7. 10.1073/pnas.2035019100 14530407 PMC218785

[66] Walker MP Brakefield T Morgan A Hobson JA Stickgold R. Practice with sleep makes perfect: sleep-dependent motor skill learning. Neuron. (2002) 35:205–11. 10.1016/S0896-6273(02)00746-8 12123620

[67] Adi-Japha E Karni A Parnes A Loewenschuss I Vakil E. A shift in task routines during the learning of a motor skill: group-averaged data may mask critical phases in the individuals' acquisition of skilled performance. J Exp Psychol Learn Mem Cogn. (2008) 34:1544–51. 10.1037/a0013217 18980413

[68] Boutin A Massen C Heuer H. Modality-specific organization in the representation of sensorimotor sequences. Front Psychol. (2013) 4:937: 10.3389/fpsyg.2013.00937 24376432 PMC3858678

[69] Diedrichsen J Kornysheva K. Motor skill learning between selection and execution. Trends Cogn Sci. (2015) 19:227–33. 10.1016/j.tics.2015.02.003 25746123 PMC5617110

[70] Oldfield RC. The assessment and analysis of handedness: the Edinburgh inventory. Neuropsychologia. (1971) 9:97–113. 10.1016/0028-3932(71)90067-4 5146491

[71] Buysse DJ Reynolds CF Monk TH Berman SR Kupfer DJ. The Pittsburgh sleep quality index: a new instrument for psychiatric practice and research. Psychiatry Res. (1989) 28:193–213. 10.1016/0165-1781(89)90047-4 2748771

[72] Ellis BW Johns MW Lancaster R Raptopoulos P Angelopoulos N Priest RG. The St. Mary's hospital sleep questionnaire: a study of reliability. Sleep. (1981) 4:93–7. 10.1093/sleep/4.1.93 7232974

[73] Vahdat S Darainy M Milner TE Ostry DJ. Functionally specific changes in resting-state sensorimotor networks after motor learning. J Neurosci. (2011) 31:16907–15. 10.1523/JNEUROSCI.2737-11.2011 22114261 PMC3260885

[74] Albert NB Robertson EM Miall RC. The resting human brain and motor learning. Curr Biol. (2009) 19:1023–7. 10.1016/j.cub.2009.04.028 19427210 PMC2701987

[75] Gabitov E Boutin A Pinsard B Censor N Fogel SM Albouy G. Re-stepping into the same river: competition problem rather than a reconsolidation failure in an established motor skill. Sci Rep. (2017) 7:9406. 10.1038/s41598-017-09677-1 28839217 PMC5570932

[76] Sakai K Kitaguchi K Hikosaka O. Chunking during human visuomotor sequence learning. Exp Brain Res. (2003) 152:229–42. 10.1007/s00221-003-1548-8 12879170

[77] Gabitov E Boutin A Pinsard B Censor N Fogel SM Albouy G. Susceptibility of consolidated procedural memory to interference is independent of its active task-based retrieval. PLoS ONE. (2019) 14:e0210876. 10.1371/journal.pone.0210876 30653576 PMC6336251

[78] Kuriyama K Stickgold R Walker MP. Sleep-dependent learning and motor-skill complexity. Learn Mem. (2004) 11:705–13. 10.1101/lm.76304 15576888 PMC534699

[79] Mazaika PK Hoeft F Glover GH Reiss AL Methods and software for fMRI analysis of clinical subjects. In: Human Brain Mapping Conference. San Francisco, CA (2009). 10.1016/S1053-8119(09)70238-1

[80] Holmes AP Friston KJ Generalisability, random effects and population inference. Neuroimage. (1998) 7:S754 10.1016/S1053-8119(18)31587-8

[81] Yousry TA Schmid UD Alkadhi H Schmidt D Peraud A Buettner A. Localization of the motor hand area to a knob on the precentral gyrus. A new landmark. Brain. (1997) 120:141–57. 9055804 10.1093/brain/120.1.141

[82] Whitfield-Gabrieli S Nieto-Castanon A. Conn: a functional connectivity toolbox for correlated and anticorrelated brain networks. Brain Connect. (2012) 2:125–41. 10.1089/brain.2012.0073 22642651

[83] Behzadi Y Restom K Liau J Liu TT. A component based noise correction method (CompCor) for BOLD and perfusion based fMRI. Neuroimage. (2007) 37:90–101. 10.1016/j.neuroimage.2007.04.042 17560126 PMC2214855

[84] Tzourio-Mazoyer N Landeau B Papathanassiou D Crivello F Etard O Delcroix N. Automated anatomical labeling of activations in SPM using a macroscopic anatomical parcellation of the MNI MRI single-subject brain. Neuroimage. (2002) 15:273–89. 10.1006/nimg.2001.0978 11771995

[85] Kieliba P Madugula S Filippini N Duff EP Makin TR. Large-scale intrinsic connectivity is consistent across varying task demands. PLoS ONE. (2019) 14:e0213861. 10.1371/journal.pone.0213861 30970031 PMC6457563

[86] Deng L Sun J Cheng L Tong S. Characterizing dynamic local functional connectivity in the human brain. Sci Rep. (2016) 6:1–13. 10.1038/srep26976 27231194 PMC4882585

[87] Tomasi D Wang R Wang GJ Volkow ND. Functional connectivity and brain activation: a synergistic approach. Cereb Cortex. (2014) 24:2619–29. 10.1093/cercor/bht119 23645721 PMC4229895

[88] Ponce-Alvarez A Thiele A Albright TD Stoner GR Deco G. Stimulus-dependent variability and noise correlations in cortical MT neurons. Proc Natl Acad Sci USA. (2013) 110:13162–7. 10.1073/pnas.1300098110 23878209 PMC3740826

[89] Maynard EM Hatsopoulos NG Ojakangas CL Acuna BD Sanes JN Normann RA. Neuronal interactions improve cortical population coding of movement direction. J Neurosci. (1999) 19:8083–93. 10.1523/JNEUROSCI.19-18-08083.1999 10479708 PMC6782478

[90] Jeanne JM Sharpee TO Gentner TQ. Associative learning enhances population coding by inverting interneuronal correlation patterns. Neuron. (2013) 78:352–63. 10.1016/j.neuron.2013.02.023 23622067 PMC3641681

[91] Sanes JN Donoghue JP Thangaraj V Edelman RR Warach S. Shared neural substrates controlling hand movements in human motor cortex. Science. (1995) 268:1775–7. 10.1126/science.7792606 7792606

[92] Toni I Krams M Turner R Passingham RE. The time course of changes during motor sequence learning: a whole-brain fMRI study. Neuroimage. (1998) 8:50–61. 10.1006/nimg.1998.0349 9698575

[93] Cramer SC Weisskoff RM Schaechter JD Nelles G Foley M Finklestein SP. Motor cortex activation is related to force of squeezing. Hum Brain Mapp. (2002) 16:197–205. 10.1002/hbm.10040 12112762 PMC6871791

[94] Penfield W Boldrey E Somatic motor and sensory representation in the cerebral cortex of man as studied by electrical stimulation. Brain. (1937) 60:389–443. 10.1093/brain/60.4.389

[95] Lv Y Margulies DS Villringer A Zang YF. Effects of finger tapping frequency on regional homogeneity of sensorimotor cortex. PLoS ONE. (2013) 8:e64115. 10.1371/journal.pone.0064115 23696867 PMC3655932

[96] Rao SM Bandettini PA Binder JR Bobholz JA Hammeke TA Stein EA. Relationship between finger movement rate and functional magnetic resonance signal change in human primary motor cortex. J Cereb Blood Flow Metab. (1996) 16:1250–4. 10.1097/00004647-199611000-00020 8898698

[97] Lehéricy S Bardinet E Tremblay L Van de Moortele P-F Pochon J-B Dormont D. Motor control in basal ganglia circuits using fMRI and brain atlas approaches. Cereb Cortex. (2006) 16:149–61. 10.1093/cercor/bhi089 15858164

[98] Orban P Peigneux P Lungu O Albouy G Breton E Laberenne F. The multifaceted nature of the relationship between performance and brain activity in motor sequence learning. Neuroimage. (2010) 49:694–702. 10.1016/j.neuroimage.2009.08.055 19732838

[99] Kwon S Watanabe M Fischer E Bartels A. Attention reorganizes connectivity across networks in a frequency specific manner. Neuroimage. (2017) 144:217–26. 10.1016/j.neuroimage.2016.10.014 27732887

[100] Povel DJ Collard R. Structural factors in patterned finger tapping. Acta Psychol. (1982) 52:107–23. 10.1016/0001-6918(82)90029-4 7164844

[101] Rosenbaum DA Kenny SB Derr MA. Hierarchical control of rapid movement sequences. J Exp Psychol Hum Percept Perform. (1983) 9:86–102. 10.1037//0096-1523.9.1.86 6220126

[102] Gerloff C Cohen LG Floeter MK Chen R Corwell B Hallett M. Inhibitory influence of the ipsilateral motor cortex on responses to stimulation of the human cortex and pyramidal tract. J Physiol. (1998) 510:249–59. 10.1111/j.1469-7793.1998.249bz.x 9625881 PMC2231019

[103] Sohn YH Jung HY Kaelin-Lang A Hallett M. Excitability of the ipsilateral motor cortex during phasic voluntary hand movement. Exp Brain Res. (2003) 148:176–85. 10.1007/s00221-002-1292-5 12520405

[104] Gorsler A Bäumer T Weiller C Münchau A Liepert J. Interhemispheric effects of high and low frequency rTMS in healthy humans. Clin Neurophysiol. (2003) 114:1800–7. 10.1016/S1388-2457(03)00157-3 14499741

[105] Kobayashi M Hutchinson S Théoret H Schlaug G Pascual-Leone A. Repetitive TMS of the motor cortex improves ipsilateral sequential simple finger movements. Neurology. (2004) 62:91–8. 10.1212/WNL.62.1.91 14718704

[106] Kobayashi M Théoret H Pascual-Leone A. Suppression of ipsilateral motor cortex facilitates motor skill learning. Eur J Neurosci. (2009) 29:833–6. 10.1111/j.1460-9568.2009.06628.x 19200062 PMC2771229

[107] Romei V Thut G Ramos-Estebanez C Pascual-Leone A M1 contributes to the intrinsic but not the extrinsic components of motor-skills. Corftex. (2009) 45:1058–64. 10.1016/j.cortex.2009.01.003 19243742

[108] Rizzo V Siebner HS Morgante F Mastroeni C Girlanda P Quartarone A. Paired associative stimulation of left and right human motor cortex shapes interhemispheric motor inhibition based on a Hebbian mechanism. Cereb Cortex. (2009) 19:907–15. 10.1093/cercor/bhn144 18791179

[109] Shim JK Kim SW Oh SJ Kang N Zatsiorsky VM Latash ML. Plastic changes in interhemispheric inhibition with practice of a two-hand force production task: a transcranial magnetic stimulation study. Neurosci Lett. (2005) 374:104–8. 10.1016/j.neulet.2004.10.034 15644273 PMC2826973

[110] Ragert P Camus M. Modulation of effects of intermittent theta burst stimulation applied over primary motor cortex (M1) by conditioning stimulation of the opposite M1. J Neurophysiol. (2009) 102:766–73. 10.1152/jn.00274.2009 19474173 PMC2724345

[111] Perez MA Wise SP Willingham DT Cohen LG. Neurophysiological mechanisms involved in transfer of procedural knowledge. J Neurosci. (2007) 27:1045–53. 10.1523/JNEUROSCI.4128-06.2007 17267558 PMC6673204

[112] Lee M Hinder MR Gandevia SC Carroll TJ. The ipsilateral motor cortex contributes to cross-limb transfer of performance gains after ballistic motor practice. J Physiol. (2010) 588:201–12. 10.1113/jphysiol.2009.183855 19917563 PMC2821559

[113] Rokni U Richardson AG Bizzi E Seung HS. Motor learning with unstable neural representations. Neuron. (2007) 54:653–66. 10.1016/j.neuron.2007.04.030 17521576

[114] Komiyama T Sato TR O'Connor DH Zhang Y-X Huber D Hooks BM. Learning-related fine-scale specificity imaged in motor cortex circuits of behaving mice. Nature. (2010) 464:1182–6. 10.1038/nature08897 20376005

[115] Kargo WJ Nitz DA. Early skill learning is expressed through selection and tuning of cortically represented muscle synergies. J Neurosci. (2003) 23:11255–69. 10.1523/JNEUROSCI.23-35-11255.2003 14657185 PMC6741030

[116] Luppino G Matelli M Camarda R Rizzolatti G. Corticocortical connections of area F3 (SMA-Proper) and area F6 (Pre-SMA) in the macaque monkey. J Comp Neurol. (1993) 338:114–40. 10.1002/cne.903380109 7507940

[117] Aizawa H Tanji J. Corticocortical and thalamocortical responses of neurons in the monkey primary motor cortex and their relation to a trained motor task. J Neurophysiol. (1994) 71:550–60. 10.1152/jn.1994.71.2.550 8176424

[118] Rouiller EM Babalian A Kazennikov O Moret V Yu X-H Wiesendanger M. Transcallosal connections of the distal forelimb representations of the primary and supplementary motor cortical areas in macaque monkeys. Exp Brain Res. (1994) 102:227–43. 10.1007/BF00227511 7705502

[119] Orgogozo JM Larsen B. Activation of the supplementary motor area during voluntary movement in man suggests it works as a supramotor area. Science. (1979) 206:847–50. 10.1126/science.493986 493986

[120] Roland PE Larsen B Lassen NA Skinhoj E. Supplementary motor area and other cortical areas in organization of voluntary movements in man. J Neurophysiol. (1980) 43:118–36. 10.1152/jn.1980.43.1.118 7351547

[121] Tanji J Okano K Sato KC. Relation of neurons in the nonprimary motor cortex to bilateral hand movement. Nature. (1987) 327:618–20. 10.1038/327618a0 3600757

[122] Bonini F Burle B Liegeois-Chauvel C Regis J Chauvel P Vidal F. Action monitoring and medial frontal cortex: leading role of supplementary motor area. Science. (2014) 343:888–91. 10.1126/science.1247412 24558161

[123] Mushiake H Inase M Tanji J. Selective coding of motor sequence in the supplementary motor area of the monkey cerebral cortex. Exp Brain Res. (1990) 82:208–10. 10.1007/BF00230853 2257906

[124] Tanji J Shima K. Role for supplementary motor area cells in planning several movements ahead. Nature. (1994) 371:413–6. 10.1038/371413a0 8090219

[125] Shima K Tanji J. Both supplementary and presupplementary motor areas are crucial for the temporal organization of multiple movements. J Neurophysiol. (1998) 80:3247–60. 10.1152/jn.1998.80.6.3247 9862919

[126] Wymbs NF Grafton ST. Contributions from the left PMd and the SMA during sequence retrieval as determined by depth of training. Exp Brain Res. (2013) 224:49–58. 10.1007/s00221-012-3287-1 23283418 PMC3539248

[127] Solopchuk O Alamia A Dricot L Duque J Alexandre Z cTBS disruption of the supplementary motor area perturbs cortical sequence representation but not behavioural performance. Neuroimage. (2017) 163:34–40. 10.1016/j.neuroimage.2017.09.013 28899743

[128] Wiestler T Waters-Metenier S Diedrichsen J. Effector-independent motor sequence representations exist in extrinsic and intrinsic reference frames. J Neurosci. (2014) 34:5054–64. 10.1523/JNEUROSCI.5363-13.2014 24695723 PMC3972728

[129] Gerloff C Corwell B Chen R Hallett M Cohen LG. Stimulation over the human supplementary motor area interferes with the organization of future elements in complex motor sequences. Brain. (1997) 120:1587–602. 10.1093/brain/120.9.1587 9313642

[130] Pinsard B Boutin A Gabitov E Lungu O Benali H Doyon J. Consolidation alters motor sequence-specific distributed representations. Elife. (2019) 8:e39324. 10.1101/376053 30882348 PMC6461441

[131] Khambhati AN Sizemore AE Betzel RF Bassett DS. Modeling and interpreting mesoscale network dynamics. Neuroimage. (2018) 180:337–49. 10.1016/j.neuroimage.2017.06.029 28645844 PMC5738302

[132] Mill RD Ito T Cole MW. From connectome to cognition: the search for mechanism in human functional brain networks. Neuroimage. (2017) 160:124–39. 10.1016/j.neuroimage.2017.01.060 28131891 PMC5529276

[133] Buckner RL Krienen FM Yeo BTT. Opportunities and limitations of intrinsic functional connectivity MRI. Nat Neurosci. (2013) 16:832–7. 10.1038/nn.3423 23799476

